# Perioperative Complications in Patients with Systemic Sclerosis: A Comparative Cohort Analysis

**DOI:** 10.18103/mra.v11i10.4606

**Published:** 2023-10-25

**Authors:** Zyad J. Carr, Luying Yan, N. David Yanez, Robert B. Schonberger, Manuel Bohorquez, Zili He, Fangyong Li, Roberta L Hines, Miriam M. Treggiari

**Affiliations:** 1 Yale University School of Medicine, Department of Anesthesiology, New Haven, Connecticut; 2 Yale New Haven Hospital, New Haven, Connecticut; 3 Yale School of Public Health, Center for Analytical Sciences, New Haven, Connecticut; 4 Duke University School of Medicine, Durham, North Carolina

**Keywords:** Systemic sclerosis, airway management, perioperative outcomes, postoperative complications, pulmonary hypertension, postoperative pneumonia

## Abstract

**Background:**

Systemic sclerosis (SSc) is a rare autoimmune disorder with pathological manifestations affecting multiple organ systems. Few studies have examined perioperative outcomes in patients with this disorder. The primary aim of this retrospective single-center comparative cohort analysis was to estimate the incidence of select perioperative complications in a population of SSc patients. In an exploratory analysis, we analyzed the relationship between SSc and susceptibility to select perioperative complications when treated at a large quaternary-care institution.

**Methods:**

We conducted a single-center retrospective, comparative cohort study to compare perioperative outcomes in a SSc (n=258) and a frequency matched control cohort (n=632). We analyzed for the presence of major composite infection (MCI), major adverse cardiac events (MACE), 30-day readmission, 30-day mortality, in-hospital complications, length of stay and airway management outcomes.

**Results:**

MCI was higher in the SSc compared to the control cohort [adjusted odds ratio (ORadj)=5.02 (95%CI: 2.47-10.20) p<0.001]. Surgical site infection (3.5% vs. 0%, p<0.001), and other infection types (5% vs. 0%, p<0.001) were higher in the SSc cohort. MACE was not significantly different between SSc vs. Control groups [6.2% vs. 7.9%, ORadj=1.33 (95%CI: 0.61-2.91) p=0.48]. Higher rates of limited cervical range of motion (13.6% vs. 3.5%, p<0.001), microstomia (11.5% vs. 1.3%, p<0.001) and preoperative difficult airway designation (8.7% vs. 0.5%, p<0.001) were observed in the SSc cohort. Bag mask ventilation grade was similar between groups (p=0.44). After adjustment, there was no between-group difference in Cormack-Lehane grade 3 and 4 view on direct laryngoscopy in SSc patients [ORadj = 1.86 (95%CI: 0.612 −5.66) p=0.18] but evidence of higher rates of video laryngoscopy [ORadj= 1.87 (95%CI:1.07 - 3.27) p=0.03]. Length of stay [median: 0.2 vs. 0.3 days, p=0.08], 30-day mortality [1.2% vs. 0.6%, ORadj=2.79 (95%CI: 0.50-15.6) p=0.24] and readmission [11.5% vs. 8.1%, ORadj=1.64 (95%CI: 0.96 - 2.82) p=0.07] were not statistically significant.

**Conclusions:**

SSc patients demonstrate mostly similar rates of MACE, 30-day mortality, length of stay intraoperative and airway complications. There is evidence of increased risk of overall 30-day MCI risk and readmission after endoscopic procedures.

## Introduction

Systemic sclerosis or scleroderma (SSc) is a rare immune-mediated disease characterized by cutaneous and organ-based fibrosis with an overall incidence of approximately 20 per million per year in the United States and a global prevalence of 150-300 cases per million^1^. Likely precipitated by complex environmental and genetic triggers, it is believed that injury to endothelial cells results in dysfunctional activation of fibroblasts, abnormal extracellular matrix deposition, and scarring in the integumentary and other organ systems^2^. Diagnosis is complex and based on clinical, radiological, and biomarker findings on a weighted scale with major criteria centered on findings of proximal cutaneous sclerosis and minor criteria including nail fold capillary pattern, the presence of pulmonary fibrosis, and SSc selective autoantibodies^3^. The four major variants of SSc are diffuse cutaneous, limited cutaneous SSc, overlap syndrome, and sine.

Limited cutaneous SSc also includes the subset of patients with Calcifications, Raynaud’s Phenomenon, Esophageal Hypomotility, Sclerodactyly, and Telangiectasia (CREST) syndrome. Diffuse cutaneous SSc is characterized by proximal limb involvement and higher rates of interstitial lung disease (ILD), and renal and cardiac involvement. All variants of SSc can result in a wide range of progressive end-organ dysfunction that carries significant perioperative implications. SSc patients may present with microstomia or limited cervical extension, with intermittent reports of difficulties with airway management.^4,5^ Autonomic dysfunction is common in SSc, and we previously identified reports of perioperative dysrhythmias in the literature 6. SSc-related myocardial fibrosis may increase the risk for myocardial infarction in the perioperative time period^7^. ILD, pulmonary hypertension (PH), and pulmonary arterial hypertension (PAH) are frequent complications of SSc^8^. Gastrointestinal involvement results in high rates of gastroesophageal reflux disease, esophageal dysmotility, dysphagia, and malnutrition^9–11^.

There is a paucity of studies that have explored the perioperative outcomes of patients with SSc. The primary aim of this retrospective single-center comparative cohort analysis was to estimate the incidence of perioperative complications in a population of SSc patients. Secondly, we sought to explore the relationship between SSc and susceptibility to select perioperative complications when treated at a large quaternary-care institution. Our outcomes included the presence of a 30-day composite measure of major postoperative infection (MCI), 30-day major adverse cardiovascular events (MACE), 30-day readmission, 30-day mortality, length of stay (LOS), intraoperative complications and airway management outcomes.

## Methods

### STUDY DESIGN

We conducted a matched cohort study using patients undergoing surgery at Yale-New Haven Hospital, a non-profit, 1,541-bed tertiary medical center located in New Haven, Connecticut, USA. This single-center retrospective study (Study #200028645) was approved by the Yale University School of Medicine Institutional Review Board with a waiver of informed consent. This manuscript adheres to the Strengthening the Reporting of Observational Studies in Epidemiology (STROBE) Statement guidelines for observational studies in epidemiology^12^.

The study cohort consisted of adult patients undergoing elective surgical or endoscopic procedures at YNHH between January 1, 2010, and January 1, 2020. Inclusion criteria for the study cohort included all patients aged >18 years old with the diagnosis of SSc based on the International Classification of Disease (ICD-10). The following ICD-10 codes were used: M34, M34.0, M34.1, M34.2, M34.8, M34.81, M34.82, M34.83, M34.83, M34.89 and M34.9 to initially screen for SSc patients. After screening by ICD-10 coding, individual charts were manually reviewed to confirm the rheumatologist’s diagnosis of systemic sclerosis (diffuse cutaneous, limited cutaneous, overlap syndrome, and sine variants only)^3^. Using frequency matching, a control cohort without the diagnosis of SSc was extracted with similar age, gender, and procedure. The final analysis included the first surgical encounter of 632 control subjects and 258 SSc subjects.

We extracted demographical data including age, gender, ethnicity, body mass index (BMI), American Society of Anesthesiologists Physical Status (ASA 1-4), and associated Elixhauser composite co-morbidities^13^.

Pulmonary hypertension (PH) was defined as a pulmonary artery mean pressure of >25mmHg by right heart catheterization within 2 years of the procedure. Elevated right ventricular systolic pressure (eRVSP) was defined as >40mmHg by echocardiography within 1year of the procedure. Procedures were categorized by anatomical location and defined as major, or minor based on the following standardized criteria. Major surgery was defined as a surgical procedure with deep tissue, abdominal, thoracic, or cranial surgical penetration, and minor surgery was defined as minimally invasive or superficial procedures.

We included gastroenterology endoscopic procedures (esophogastroduodenoscopy, colonoscopy, endoscopic retrograde cholangiopancreatography). Surgical encounter characteristics were classified into 16 individual anatomical categories (Head/neck major and minor, thoracic major and minor, spine/spinal cord major, upper/lower abdomen major, urologic/gynecologic major, hip/ leg/ shoulder /arm /hand major/minor, endoscopy, cardiac major/minor, obstetrics, vascular major/minor).

Anaesthesia type was characterized into nine categories (general endotracheal anaesthesia, general laryngeal mask anaesthesia, neuraxial anaesthesia, regional anaesthesia only, regional anaesthesia and general endotracheal anaesthesia (ETT), regional anaesthesia and general laryngeal mask airway (LMA), general anaesthesia with a natural airway, conscious sedation, and not recorded). Preoperative anaesthesia data included Mallampati airway class (1-4), thyromental distance (< or >3 cm), neck range of motion (normal vs. limited), presence of documented microstomia (<3.5cm; yes/no) and pre- and postoperative difficult designation (yes/no). Airway management data extracted included mask ventilation grade [Grade 0: not attempted, Grade 1: ventilated by mask; Grade 2: ventilated by mask with oral airway or other adjuvant; Grade 3: difficult mask ventilation (inadequate, unstable, or requiring 2 practitioners, 4 unable to mask ventilate)], presence of rapid sequence intubation technique (yes/no), direct (DL) or video laryngoscopy (VL) attempts and graded view (Cormack-Lehane Grade I - IV), presence of fiberoptic bronchoscopy (yes/no), unanticipated postoperative difficult airway designation (yes/no), presence of failed intubation (yes/no), and airway or dental injury 14.

Intraoperative adverse events included arterial oxygen desaturation (Sp02<88%) within 15 minutes of induction, hypotension (systolic BP <90 mmHg or mean arterial pressure <60mmHg) within 15 minutes of induction, intraoperative reintubation, intraoperative cardiac arrest, tachycardia (>100 beats per minute) within 15 minutes of induction, bradycardia (<60 beats per minute) within 15 minutes of induction, intraoperative vasopressor use (infusion or ≥1 recorded instance of ephedrine, phenylephrine, epinephrine, norepinephrine, or vasopressin administration), presence of cardiac arrhythmia, intraoperative bronchospasm, anaphylaxis, and pulmonary aspiration.

We collected select in-hospital respiratory-related complications (composite reintubation, non-invasive positive pressure ventilation, mechanical ventilation, and intensive care admission) and LOS (in days). 30-day postoperative complications were comprised of: major adverse cardiovascular events (MACE; 30-day myocardial infarction, stroke or transient ischemic attack, atrial fibrillation and congestive heart failure), Major Composite Infection [MCI; defined as: 30-day postoperative pneumonia, aspiration pneumonia, surgical site infection or other infection (other infection: 30-day urinary tract infection, bloodstream infection or sepsis, unspecified)], 30-day deep vein thrombosis/pulmonary embolism (DVT/PE), 30-day readmission and 30-day mortality.

The analyzed outcomes of the study include the presence of MCI, MACE, 30-day readmission, 30-day mortality, composite in-hospital respiratory-related complications, LOS, and airway management outcomes (DL vs. VL, presence of grade Cormack-Lehane Grade III/IV view).

### STATISTICAL ANALYSIS

We summarized patient characteristics using means and standard deviations (SD) and/or median and interquartile range (IQR) for continuous variables and frequencies and percentages for categorical variables. Baseline characteristics for SSc and control subjects were compared using chi-square tests or Fisher’s exact tests for categorical variables and t-tests or Wilcoxon Rank Sum tests for continuous variables. We visually inspected for normal distribution of the data. We removed emergency procedures from the control and SSc cohorts prior to analysis. For the binary perioperative outcomes, multivariable logistic regression analyses were used to compare the odds of complication between cohorts and also to assess the factor of SSc with the selected outcomes after controlling for clinically relevant confounders. For the continuous perioperative outcomes, multivariable linear regression analyses were used. Confounding variables were identified by clinical experience, prior clinical validation or physiological rationale and availability. Adjustments performed for the postoperative 30-day outcomes included: Age, BMI, Elixhauser comorbidities, procedural category, ASA physical status. For airway management (Incidence of video laryngoscopy, incidence of Cormack-Lehane Classification grade 3 and 4 on direct laryngoscopy), adjustments performed included: age, BMI, ASA physical status. For the purposes of regression model stability, procedural categories were collapsed into 5 anatomically similar and clinically relevant groups. Upon collapse, no significant difference was observed between groups [p=0.71]. Statistical significance was established at 0.05 level. No correction was made for multiple comparison given the exploratory nature of the analysis. All analyses were performed using SAS version 9.47.

## Results

**Figure 1.**
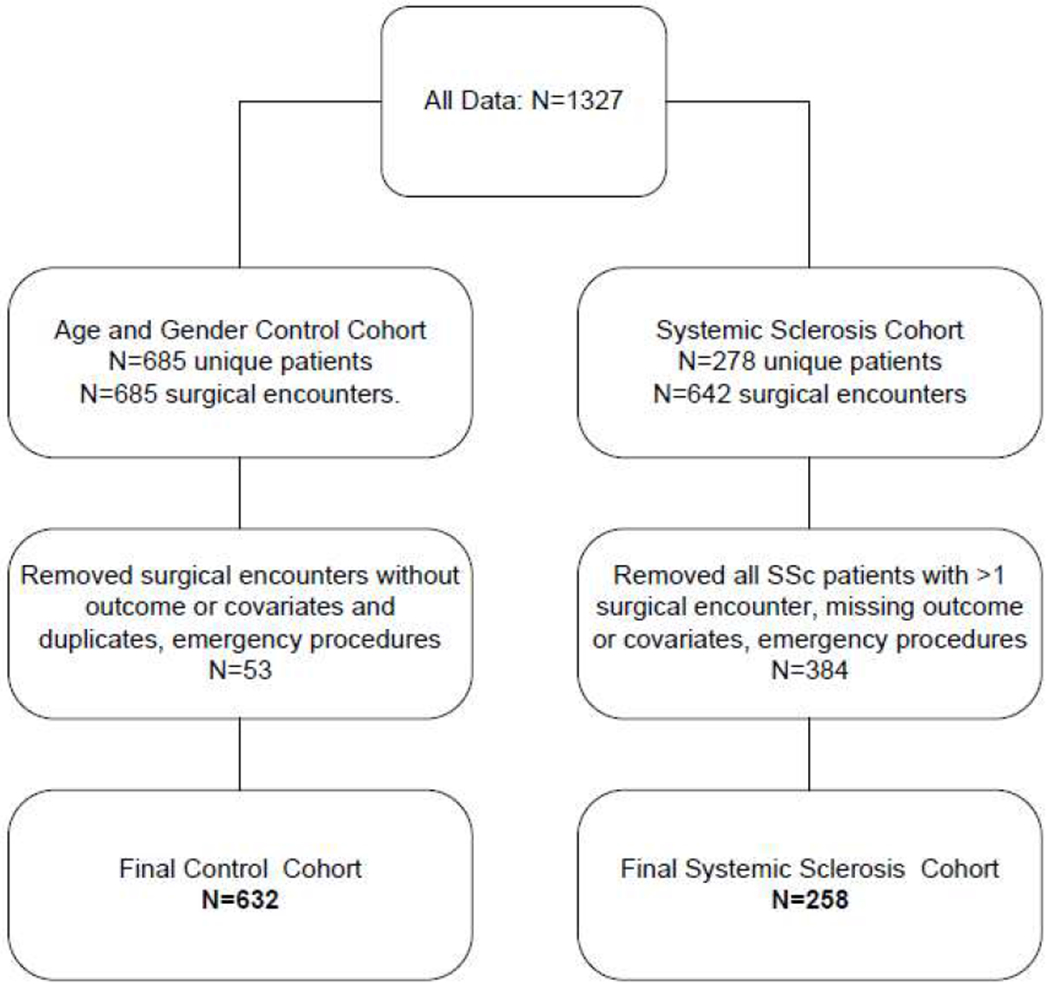
A CONSORT diagram of patient selection for the study and control cohort.

We identified 632 control patients and 258 SSc patients who underwent surgery and had follow-up for a minimum of 30 days. The CONSORT flowchart of research subject inclusion is provided in Figure 1.

Demographic data for the SSc and control cohorts are summarized in Table 1.

Detailed sub-cohort demographical data and select univariate data is provided in Supplementary Table 1. The SSc cohort was comprised of diffuse cutaneous (N=51, 19.8%), limited cutaneous (N=173, 67.1%), overlap (N=31, 12.0%) and sine (N=3, 1.2%) variants. The SSc cohort had higher ASA severity (p<0.001) but demonstrated similar age [59.8 (±14.4) vs. 59.8 (±15.5), p=0.98, SSc vs. control, respectively] and gender [female: 84.5% vs. 81.2%, p=0.24] to the control cohort. Anesthesia type was not significantly different between cohorts when collapsed into clinically relevant categories (p=0.22). Mean surgical time was shorter by approximately 19.3 minutes in the SSc cohort [94.4 (±98.89) vs. 113.7 (±100.2), p=0.01]. Also, the median and IQR surgical time in the SSc cohort were shorter [54 minutes, (IQR 32 - 124) vs 78 (44 - 155.5), p < 0.001]. A comparison of Elixhauser composite variables used for statistical adjustment is provided in Table 2.

### AIRWAY MANAGEMENT AND INTRAOPERATIVE COMPLICATIONS

Airway management and intraoperative complication characteristics for the SSc and control cohort are summarized in Table 3. Upon univariate analysis, SSc patients demonstrated higher rates of limited cervical range of motion (13.6% vs. 3.5%, p<0.001), microstomia (11.5% vs. 1.34%, p<0.001) and preoperative difficult airway designation (8.7% vs. 0.5%, p<0.001) when compared to the control cohort. Within SSc variants, microstomia was most common in the diffuse cutaneous variant [N=37 (72.5%)]. Ventilation grade was similar between SSc vs. control groups (Grade 1/2 98.3% vs. 95.3%, Grade 3/4 0.4% vs. 1.14%, p=0.44). After adjustment for age, BMI, and procedural category, there was no evidence of a significant difference in Cormack-Lehane Grade 3 or 4 views on DL in SSc patients (ORadj = 1.86, 95% CI: 0.61 −5.66, p=0.18) but evidence of higher rates of VL (30% vs. 17.4%, ORadj= 1.87, 95% CI: 1.07-3.27, p=0.03). Regarding intraoperative minor and major complications in the SSc vs. control cohort, a higher incidence of induction-related bradycardia (16.3% vs. 1.3%, p<0.001) was observed but induction-related hypotension was similar (23.5% vs. 18.2%, p=0.07). Rates of intraoperative pulmonary [pulmonary aspiration (p=0.36); induction-related arterial desaturation (p=0.66)], and cardiac complications [hypotensionrequiring vasopressor administration(p=0.36);intraoperative arrhythmia (p=0.12)] were similar between groups.

### EXPLORATORY ANALYSIS OF SELECTED PERIOPERATIVE OUTCOMES

Perioperative 30-day outcomes are described in Table 4. After adjustment for age, gender, Elixhauser covariates, and procedural category, the SSc cohort was significantly more likely to have MCI compared to the control cohort [12.8% vs. 2.7% ORadj=5.02 (95% CI: 2.47 - 10.2) p<0.001]. The results were similar when stratified by endoscopy procedure.

SSc patients demonstrated higher risk for MCI in endoscopic [ORadj=4.90 (95%CI: 2.28 - 10.54) p<0.001] and non-endoscopic procedures [ORadj=4.56 (95%CI: 1.01 - 20.8) p=0.049]. MCI events had the highest incidence in the overlap variant (N=5, 16.1%) but were also common in limited cutaneous (N=20, 11.5%) and diffuse cutaneous variants (N=4, 7.8%). SSc patients who received endoscopic procedures were more likely to be readmitted in 30 days [ORadj=1.86 (95%CI: 1.03 - 3.37) p=0.03] compared to their counterparts in the control cohort, while readmission in SSc patients undergoing non-endoscopic procedures was like those in the control cohort (p=0.99). Univariate analysis demonstrated that surgical site infection (3.54% vs. 0%, p<0.001), and other infection types (4.98% vs. 0%, p<0.001) were the composite components that were significantly higher in the SSc cohort.

Further detailed information regarding the surgical site infections is provided in Supplementary Table 2. 30-day pneumonia (3.97% vs. 1.74%, p=0.12) and aspiration pneumonitis/pneumonia (1.19% vs. 0.95%, p=0.72) appeared to be more frequent in the SSc cohort but did not reach statistical significance. After adjustment for age, gender, Elixhauser covariates and procedural category, MACE was not significantly different between SSC and control cohorts (ORadj=1.33, (95%CI: 0.61 - 2.91) p=0.48) but was higher in the SSc endoscopic cohort when compared to the control endoscopic cohort [ORadj=1.86 (95%CI: 1.03 - 3.37) p=0.03]. Postoperative in-hospital initiation of noninvasive (1.16% vs. 0%, p=0.02) and invasive (1.55% vs. 0%, p=0.007) positive pressure ventilation was higher in the SSc group. No differences were observed in 30-day mortality [ORadj=2.79, 95% CI, 0.50 - 15.6, p=0.24). Multivariable linear regression was used to test if the SSc cohort differed significantly in LOS, when adjusted for Elixhauser covariates and procedure category, but did not demonstrate significance [adjusted mean difference = −0.71 days, (95%CI: −1.63 - 0.02) p=0.13].

### EXPLORATORY SUB-COHORT ANALYSIS: ROLE OF PH AND INTERSTITIAL LUNG DISEASE

Echocardiographic and right heart catheterization data of the SSc cohort are presented in Table 5. In our SSc cohort, 8.5%% (N=22) had documented PH by right heart catheterization. Pre-capillary PH constituted 68.2% (N=15) and 31.8% (N=7) had post-capillary PH. In addition, 12% (N=31) of SSc patients had eRVSP by echocardiography, defined as RVSP >40mmHg, with a mean RVSP of 60.0 (±18.7). In SSc patients with PH, average pulmonary artery mean pressure was 39.1 (±9.91). No relationship was observed between eRVSP and 30-day pneumonia [ORadj=0.25 (95% CI: 0.002 - 41.9) p=0.6]. No relationship was observed between PH and 30-day pneumonia [ORadj= 0.41 (95% CI: 0.002-112.0) p=0.76]. Separate sub-cohort analysis of pre- and postcapillary PH did not demonstrate a statistically significant association with MCI on univariate analysis (pre-capillary PH: 20% vs. post-capillary PH: 0%, p=0.20), although this finding is limited by small sample size.

We observed an incidence of preoperative diagnosis of interstitial lung disease (ILD) of 32.9% in our SSc cohort [diffuse cutaneous: N=37 (66.7%), limited cutaneous: 37 (21.3%) overlap 13 (41.9%) sine 1 (33.3%)] but it did not appear to predict MCI when similarly adjusted for Elixhauser comorbidities, age, procedural category and gender [ILD: 5.0% vs. no ILD: 6.2%, ORadj=1.67, (95%CI: 0.71-3.92) p=0.24].

## Discussion

Our aim for this study was to contribute baseline estimates of perioperative complications in an SSc population that has been previously neglected in the literature. In our literature review, we found a number of case reports and series describing airway management difficulties, intraoperative cardiac arrest, and other perioperative adverse events that warranted larger scale analysis^4,15–20^.

Data on 30-day perioperative complications, airway management, and intraoperative complications in SSc patients are, to our knowledge, not readily available in the literature. Microstomia (1.3% vs. 11.5%, p<0.001) and limited cervical range of motion (3.5% vs. 13.6%, p<0.001) were both very common in the SSc cohort. Microstomia has been previously identified as an important cause for difficult airway but was only documented in 11.5% of our SSc cohort. Microstomia is reported to affect 52-80% of SSc patients, suggesting that it may be under-recognized at the time of preoperative physical examination^21,22^. Incidence of difficult mask ventilation (Grade 3 or 4) was not significantly different between cohorts (1.1% vs. 3.1%, p=0.44). Although direct laryngoscopy first-pass success was lower in the SSc cohort (91.8% vs. 95.8%, p=0.18) it was not statistically significant. We analyzed VL use and found a significantly higher incidence of use in SSc patients when compared to controls. Increased VL use (30% vs. 17.4%, p=0.027) and similar rates of failed intubation (1.1% vs. 0%, p=1), suggest that preoperative identification of potential difficult airway and pre-emptive use of VL may have reduced the excess risk of difficult airway instrumentation in SSc patients^23^.

Intraoperative complications were similar between cohorts with the exception of induction-related bradycardia (16.3% vs. 1.3%, p<0.0001). This is an interesting finding and may be associated with case reports of cardiac arrest related to sinoatrial arrest observed in the literature. Fortunately, in our SSc cohort, these bradycardic events resolved uneventfully, and intraoperative cardiac arrests (0% vs. 1%, p=1) and intraoperative cardiac arrhythmia (2.3% vs. 0.9%, p=0.12) were similar between cohorts ^24,25^. This also may suggest that the current management of SSc-related cardiac conditions are well managed prior to presentation for surgery^25^.

In our exploratory analysis, we were able to report that a composite infection measure using meaningful grouping, MCI, was significantly higher in postoperative SSc patients when compared to a control cohort. This significance was predominately contributed by the development of higher rates of 30-day postoperative surgical site infection, urinary tract infection, bloodstream infection or sepsis when compared to controls. SSc patients with skin ulcers secondary to microcirculatory vasculopathy have been perceived to have higher rates of impaired wound healing^26–28^. Secondly, all-cause mortality studies in SSc populations have observed that 33% of deaths due to non-SSc-related causes were attributed to infections, particularly septicemia and pneumonia^29^. Thus, our observation of higher postoperative infection is likely consistent with prior findings of increased infection risk in SSc populations. Due to the rarity of the disorder, the SSc cohort was not adequately powered to provide observations into individual respiratory complications though a higher incidence was observed in 30-day postoperative pneumonia (1.7% vs. 4.0% p=0.12). Surprisingly, despite the common presence of esophageal dysmotility disorders in SSc, no statistically significant relationship was observed in intraoperative aspiration (0.5% vs. 1.2%, p=0.36) nor 30-day aspiration pneumonitis/pneumonia (1% vs. 1.2%, p=0.72) in our SSc cohort. However, MCI and readmission was higher in SSc compared to controls after endoscopic procedures, largely driven by a larger incident in other infection (urinary tract infection, bloodstream infection or unspecified sepsis). This may suggest that infectious complications are being underdiagnosed after endoscopic procedures in these high-risk patients. Overall, the SSc cohort’s higher rate of postoperative infection suggest that improved post-procedure vigilance and a higher level of suspicion for infection is warranted.

Luo et al. did not observe an association between perioperative MACE but did present evidence suggestive that perioperative myocardial infarction may be higher in SSc populations^7^. We similarly observed that MACE was not statistically different between control and SSc cohorts. eRVSP (12.0%) and diagnosed PH (8.5%) were relatively common in our SSc cohort. This is consistent with the published prevalence of pulmonary hypertension in SSc populations (5-12%) but a much higher prevalence when compared to the general surgical population (0.81% for noncardiac surgery, 10.72% for cardiac surgery)^30–32^. Furthermore, the presence of eRVSP has been observed to increase overall mortality^33^. SSc-related PH has demonstrated lower response rates to targeted therapy and poorer prognosis, thus identifying and optimizing these patients may improve perioperative outcomes^34^. Furthermore, we were interested in the association of PH or eRVSP and 30-day pneumonia given recent data that has suggested a pathophysiological relationship between dysregulated immune responses in pulmonary hypertension and increased risks of respiratory complications^35,36^. Although 30-day pneumonia was higher in the SSc cohort, sub-cohort analysis of eRVSP and PH SSc sub-cohorts failed to achieve statistical significance, but this finding is limited by small sample size.

The possibility of selection bias exists, given the retrospective and exploratory nature of the study. Furthermore, as a retrospective study, these observations can only provide association, rather than causation. We cannot rule out the possibility that unmeasured confounding affected our findings, particularly changing medical practice over the decade of data collection. Given the complexity of SSc diagnoses, we were heavily reliant on rheumatologist expertise for inclusion during manual chart review of ICD-10 identified research subjects. In regard to observations of airway management, it is important to acknowledge that significant inter-proceduralist variability exist regarding standard of care and technique. We acknowledge that this increases the likelihood of type II error, and that multiple testing increases the probability of type I errors in our observations. We utilized a composite measure of highly related and common postoperative infectious and cardiovascular endpoints to better control for Type I errors in our statistical analysis.

## Conclusion

In conclusion, we provided detailed estimates of the incidence of perioperative complications within a large cohort of SSc patients. First-pass success with direct laryngoscopy appears to be statistically similar to control cohort and there is no significant increased frequency of difficult bag mask ventilation. We found that VL use is more frequent in SSc and has possibly contributed to reduced airway instrumentation risk in this population. We observed significantly higher incidences of induction-related bradycardia in our SSc cohort, but this did not lead to a statistically significant increased risk of persistent intraoperative cardiac arrhythmia or arrest when compared to the control population. Secondly, we observed that a 30-day composite measure of infection (MCI) was significantly higher when compared to a control cohort although 30-day MACE, 30-day mortality, readmission and length of stay were similar to the control cohort. This observed higher risk of 30-day perioperative infection should be considered during perioperative care of SSc patients. To improve the precision of these exploratory findings, further corroboration with larger multi-center cohorts is warranted.

## Supplementary Material

1

## Figures and Tables

**Table 1:** Patient Demographical and clinical characteristics of the SSc (N=258) and control cohorts (N=632)

Characteristics *	Control	SSc	P value
Age, years	59.8 ± 15.5	59.8 ± 14.4	0.98
			
Age groups			0.74
≤ 45 years	113 (17.9%)	44 (17.1%)	
46 - 60 years	177 (28.0%)	80 (31.0%)	
61 - 70 years	172 (27.2%)	72 (27.9%)	
>70 years	170 (26.9%)	62 (24.0%)	
			
BMI**, kg/m^2^	29.2 ± 7.9	26.7 ± 6.5	**< 0.001**
BMI groups			0.006
Normal	172 (27.2%)	93 (36.1%)	
Underweight	16 (2.5%)	11 (4.3%)	
Overweight	153 (24.2%)	61 (23.6%)	
Class 1 obesity	103 (16.3%)	31 (12.0%)	
Class 2/3 obesity	109 (17.3%)	25 (9.7%)	
Not recorded	79 (12.5%)	37 (14.3%)	
Gender			0.24
Female	513 (81.2%)	218 (84.5%)	
Male	119 (18.8%)	40 (15.5%)	
ASA Classification†			**<0.001**
1 and 2	293 (46.3%)	85 (32.94%)	
3 and 4	320 (50.6%)	173 (67.05%)	
Not Recorded	19 (3.0%)	0 (0%)	
Procedural Category‡			**<0.001**
Head/neck major	20 (3.2%)	21 (8.1%)	
Head/neck minor	42 (6.7%)	11 (4.3%)	
Thoracic major	5 (0.8%)	4 (1.6%)	
Thoracic minor	33 (5.2%)	12 (4.7%)	
Spine/spinal cord major	22 (3.5%)	15 (5.8%)	
Upper and lower abdomen major	46 (7.3%)	27 (10.5%)	
Urologic/gynecologic/pelvis major	48 (7.6%)	13 (5.0%)	
Hip/leg/shoulder/arm/hand major	56 (8.9%)	19 (7.4%)	
Hip/leg/shoulder/arm/hand minor	58 (9.2%)	20 (7.8%)	
Endoscopy	208 (32.9%)	90 (34.9%)	
Cardiac Major	14 (2.2%)	9 (3.5%)	
Cardiac Minor	27 (4.3%)	3 (1.2%)	
Obstetrics	11 (1.7%)	1 (0.4%)	
Interventional radiology	27 (4.3%)	1 (0.4%)	
Vascular Major	3 (0.5%)	3 (1.2%)	
Vascular Minor	2 (0.3%)	3 (1.2%)	
Neurosurgery major	7 (1.1%)	4 (1.6%)	
Transplant major	3 (0.5%)	2 (0.8%)	
Anesthesia Category			0.22
General endotracheal anesthesia	224 (35.4%)	90 (34.9%)	
General laryngeal mask anesthesia	74 (11.7%)	24 (9.3%)	
Neuraxial anesthesia	32 (5.1%)	10 (3.9%)	
Regional anesthesia only	0 (0.00%)	5 (1.9%)	
Regional anesthesia and general anesthesia	11 (1.7%)	6 (2.3%)	
General, natural airway	221 (35.0%)	103 (39.9%)	
Conscious sedation	64 (10.1%)	16 (6.2%)	
Not Recorded	6 (0.9%)	4 (1.6%)	
Clinical Outcomes			
Length of Stay Mean, in days, mean (SD)	2.6 (±5.6)	2.1 (±5.6)	0.22
Length of Stay Median, in days, median (IQR)	0.3 (0.1-49.2)	0.2 (0.1-49.9)	0.08
30-day Mortality	4 (0.6%)	3 (1.2%)	0.41
30-day Readmission	51 (8.1%)	29 (11.5%)	0.11

* Mean ± standard deviation for continuous variables and n (column %) for categorical variables.

† American Society of Anesthesiologists- Physical Status

‡ For the purposes of statistical analysis, these 18 categories were condensed into 5 categories by anatomical region, head/neck/neurological; thoracic/cardiac; abdominal; musculoskeletal/spine; endoscopic/minimally invasive (interventional radiology). There were no differences after adjustment [η^2^=(4, 890) p=0.71].

**Table 2.** Demographical Data. Selected Elixhauser covariates at time of surgical encounter

Co-morbidity n (%)	Control (N=632)	Systemic Sclerosis (N=258)	P-value
			
Neurological Disorders			
Paralysis	6 (1.0%)	1 (0.4%)	**0.02**
Neurological Disorders	19 (3.0%)	1 (0.4%)	**0.02**
Cerebrovascular Disease	27 (4.3%)	5 (1.9%)	0.09
Transient Ischemic Attack	0 (0%)	0 (0%)	1
Intracranial Hemorrhage	3 (0.5%)	0 (0.0%)	0.56
Stroke Disorder	8 (1.3%)	0 (0.0%)	0.11
			
Cardiovascular Disorders			
Lipid Disorder	107 (16.9%)	26 (10.1%)	**0.01**
Hypertension, uncomplicated	77 (12.2%)	31 (12.0%)	0.94
Hypertension, complicated	19 (3.0%)	04 (1.6%)	0.21
Myocardial Infarction	15 (2.4%)	3 (1.2%)	0.24
Congestive Heart Failure	34 (5.4%)	9 (3.5%)	0.23
Valvular Disease Disorders	36 (5.7%)	15 (5.8%)	0.95
Pulmonary Circulatory Disorders	9 (1.4%)	6 (2.3%)	0.39
Atrial Fibrillation or Flutter	22 (3.5%)	5 (0.9%)	0.22
Bradycardia related disorders	4 (0.6%)	1 (0.4%)	1
Arrhythmia Disorders, all	54 (8.5%)	16 (6.2%)	0.24
Peripheral Vascular Disease	32 (5.1%)	17 (6.9%)	0.37
			
Psychiatric and Substance Abuse Disorders			
Depressive Disorders	37 (5.9%)	11 (4.3%)	0.34
Psychosis and Related Disorders	5 (0.8%)	0 (0%)	0.33
Ethanol Abuse Disorder	12 (1.90%)	0 (0%)	**0.02**
Drug Abuse Disorder	7 (1.1%)	2 (0.8%)	1
Nicotine Dependence	39 (6.2%)	6 (2.3%)	0.02
			
Pulmonary Disorders			
Pulmonary Disease, all	55 (8.7%)	31 (12.0%)	0.13
Chronic Obstructive Pulmonary Disease	20 (3.2%)	10 (3.9%)	0.59
Asthma Disorders	30 (4.8%)	13 (5.0%)	0.85
			
Gastrointestinal Disorders			
Peptic Ulcer Disease	8 (1.3%)	4 (1.6%)	0.75
Liver Disease	26 (4.1%)	10 (3.9%)	0.87
			
Renal Disorders			
Chronic Kidney Disease	15 (2.4%)	4 (1.6%)	0.44
End-Stage Renal Disease	3 (0.5%)	1 (0.4%)	1
Electrolyte Disorders	53 (8.4%)	18 (7.0%)	0.48
Renal Transplantation	1 (0.2%)	1 (0.4%)	0.5
			
Endocrine Disorders			
Diabetes, uncomplicated	19 (3.0%)	8 (3.1%)	0.27
Diabetes, complicated	19 (3.0%)	1 (0.4%)	0.02
Hypothyroidism	33 (5.2%)	8 (3.1%)	0.17
Obesity Disorder	55 (8.7%)	9 (3.5%)	**0.01**
			
Hematological/Oncological/Infectious Disorders			
Metastatic Disease	14 (2.2%)	3 (1.2%)	0.42
Lymphoma	3 (0.5%)	3 (1.2%)	0.36
Solid Tumor	45 (7.1%)	12 (4.7%)	0.17
Blood Loss Anemia	9 (1.4%)	2 (0.8%)	0.53
Iron Deficiency Anemia	11 (1.7%)	9 (3.5%)	0.11
Coagulopathy	16 (2.5%)	2 (0.8%)	0.09
Weight Loss	18 (2.9%)	6 (2.3%)	0.66
HIV	2 (0.2%)	0 (0%)	1

**Table 3.** Intraoperative Characteristics of the Control and Systemic Sclerosis Cohort

	Control Cohort (N=632)	SSc Cohort (N=258)	p
**Airway Characteristics n (%)** *			
Mallampati Class			0.17
1 and 2	471 (74.5%)	189 (73.3%)	
3 and 4	121 (46.9%)	67 (25.9%)	
Not Recorded	40 (6.3%)	2 (0.8%)	
Thyromental Distance			
<3 Fingerbreadths	41 (6.5%)	25 (9.7%)	0.137
>3 Fingerbreadths	554 (87.7%)	228 (88.3%)	
Not Recorded	37 (5.9%)	5 (1.9%)	
Limited Range of Motion			**<0.001**
Yes	22 (3.5%)	35 (13.6%)	
No	574 (90.8%)	219 (84.9%)	
Not Recorded	36 (5.7%)	4 (1.6%)	
Microstomia			**<0.001**
Yes	8 (1.3%)	29 (11.5%)	
No	589 (93.2%)	223 (86.4%)	
Not Recorded	36 (5.7%)	4 (1.6%)	
			
**Airway Management**			
Preoperative Difficult Airway Designation			**<0.001**
Yes	3 (0.5%)	22 (8.7%)	
No	619 (97.9%)	231 (89.5%)	
Not Recorded	10 (1.6%)	5 (1.9%)	
Ventilation Grade (% of attempted)†	224 90		0.44
Not Recorded	18 (8.0%)	5 (5.6%)	
Grade 1 or 2	144 (64.2%)	59 (65.5%)	
Grade 3 or 4	7 (3.1%)	1 (1.1%)	
Not Attempted	55 (24.6%)	25 (27.8%)	
Rapid Sequence Intubation†			0.2
Yes	45 (20.0%)	25 (27.8%)	
No	182 (81.2%)	65 (72.2%)	
Not Recorded	2 (0.9%)	0 (0%)	
Direct Laryngoscopy†			0.38
Yes	167 (74.6%)	61 (67.7%)	
No	51 (22.8%)	27 (30.0%)	
Not Recorded	6 (2.7%)	2 (2.2%)	
Direct Laryngoscopy Cormack-Lehane Grade†			0.88
Grade 1 or 2	154 (68.8%)	54 (60.0%)	
Grade 3 or 4	12 (7.23%)	6 (10.0%)	
Not Recorded	58 (25.9%)	60 (66.7%)	
Direct Laryngoscopy Attempts†			0.18
1 attempt	160 (71.4%)	56 (62.2%)	
2 or more attempts	7 (3.1%)	5 (5.6%)	
Not Recorded	57 (25.4%)	29 (32.2%)	
Video Laryngoscopy†			**0.027**
Yes	39 (17.4%)	27 (30.0%)	
No	179 (79.9%)	61 (67.8%)	
Not Recorded	6 (2.7%	2 (2.2%)	
Video Laryngoscopy Cormack-Lehane Grade			0.64
Grade 1 or 2	38 (97.4%)	26 (96.3%)	
Grade 3 or 4	1 (2.56%)	1 (3.70%)	
Not Recorded	0 (0%)	0 (0%)	
Video Laryngoscopy Attempts‡			0.64
1 attempt	35 (89.7%)	21 (77.8%)	
2 or more attempts	4 (10.26%)	1 (3.7%)	
Not Recorded	0 (0%)	5 (5.5%)	
Fiberoptic Bronchoscopy†			0.12
Yes	6 (2.7%)	6 (6.6%)	
No	218 (97.3%)	84 (93.3%)	
Not Recorded	0 (0%)	0 (0%)	
Postoperative Difficult Airway Designation†*			**0.025**
Yes	5 (2.2%)	8 (8.9%)	
No	209 (93.3%)	79 (87.8%)	
Not Recorded	10 (4.5%)	3 (3.3%)	
Airway Complications			
Failed Intubation	0 (0%)	1 (1.1%)	1
Airway Injury	0 (0%)	0 (0%)	1
Dental Injury	0 (0%)	0 (0%)	1
Anesthesia Management			
Surgical Time, time in minutes (SD)	113.7 (±100.2)	94.4 (±98.89)	**0.01**
			
**Reported Intraoperative Complications** **			
Induction			
Arterial oxygen desaturation (<88%, <10min)	14 (2.2%)	7 (2.7%)	0.66
Hypotension (<10min)	115 (18.2%)	61 (23.6%)	0.07
Tachycardia (<15min)	18 (2.8%)	16 (6.2%)	0.019
Bradycardia (<15min)	8 (1.3%)	42 (16.3%)	**<0.001**
Cardiac			
Hypotension requiring vasopressors	192 (30.9%)	76 (29.5%)	0.36
Intraoperative Arrhythmia	6 (0.9%)	6 (2.3%)	0.12
Intraoperative Cardiac Arrest	1 (0.16%)	0 (0%)	1
Respiratory			
Intraoperative Reintubation	2 (0.3%)	1 (0.4%)	1
Intraoperative Bronchospasm/Laryngospasm	3 (0.5%)	0 (0%)	0.56
Intraoperative Pulmonary Aspiration	3 (0.5%)	3 (1.2%)	0.36
Immunological			
Intraoperative Anaphylaxis	0 (0%)	0 (0%)	1

* % Derived from total encounters Control N=632 and SSc cohort N=258

† % Derived from encounters marked as general endotracheal anesthesia (Control N=224 and SSc cohort N=90). Note some airway management attempts may have started with direct laryngoscopy but transitioned to indirect video laryngoscopy or fiberoptic bronchoscopy due to laryngoscopic view.

‡ % Derived from total video laryngoscopic encounters (Control; N=39 and SSc; N=27)

* Measurement of unanticipated difficult airway

** Calculated from all surgical encounters (Control=632; SSc=258); Missing data: Control cohort=10 patients SSc cohort=3 patients.

**Table 4.** Selected univariate and multivariable in-hospital and 30-day clinical outcomes.

	Control (N=632)	Systemic Sclerosis (N=258)	P Value
**In-hospital Outcomes**			
Intensive care admission	40 (6.4%)	9 (3.5%)	0.09
Reintubation	5 (0.8%)	3 (1.2%)	0.7
Noninvasive positive pressure ventilation initiation	0 (0.00%)	3 (1.2%)	0.02
Mechanical ventilation initiation	0 (0.00%)	4 (1.6%)	**0.007**
			
**30-day clinical outcomes**			

**Major Adverse Cardiovascular Events n (%)**			
Myocardial Infarction	0 (0%)	0 (0%)	1
Atrial Fibrillation	22 (3.5%)	7 (2.8%)	0.58
Congestive Heart Failure	25 (4.0%)	9 (3.5%)	0.77
Stroke	3 (0.5%)	0 (0%)	0.56
Deep Vein Thrombosis/Pulmonary Embolism	0 (0%)	1 (0.6%)	0.22
**Major 30-day Infection Outcomes n (%)**			
Surgical Site Infection	0 (0%)	9 (3.5%)	**<0.001**
Pneumonia	11 (1.7%)	10 (4.0%)	0.12
Aspiration Pneumonitis or Pneumonia	6 (1.0%)	3 (1.2%)	0.72
Other Infection Types	0 (0%)	11 (5.0%)	**<0.001**
			
**Multivariable Logistic Regression Analysis** *	**Odds Ratio**	**Confidence Interval**	**Adjusted P value**
			
MCI^†^ (SSc vs. Control)	5.02	2.47-10.20	**<0.001**
Subset Receiving Endoscopy	4.9	2.28-10.54	**<0.001**
Subset Receiving Other Procedures	4.56	1.01-20.80	**0.049**
MACE^‡^ (SSc vs. Control)	1.33	0.61-2.91	0.48
30-day Readmission (SSc vs. Control)	1.64	0.96-2.82	0.07
Subset Receiving Endoscopy	1.86	1.03-3.37	**0.03**
Subset Receiving Other Procedures	1	0.30-3.38	0.99
30-day Mortality (SSc vs. Control)	2.79	0.50-15.6	0.24
			
**Sub-cohort analysis (eRVSP vs. Other, SSc)** *			
30-day Pneumonia	0.25	0.002-41.9	0.6
Composite In-hospital Outcomes^¥^	0.67	0.05 - 9.34	0.77
**Sub-cohort analysis (PH vs. Other, SSc)** *			
30-day Pneumonia	0.41	0.002-112.0	0.76
Composite In-hospital Outcomes	4.92	0.57-42.7	0.15

**Airway Management** **			
McCormick Grade 3 and 4 (SSc vs. Control)	1.86	0.61 - 5.66	0.18
Video Laryngoscopy (SSc vs. Control)	1.87	1.07 - 3.27	**0.03**

Abbreviations: MCI - major composite infection; SSc - systemic sclerosis; eRVSP: >40mmHg right ventricular systolic pressure (by echocardiography); PH: >25 mmHg pulmonary artery systolic pressure by right heart catheterization.

† composite major infection - 30-day surgical site infection, pneumonia, aspiration pneumonitis or pneumonia, and other infection type.

‡ Major adverse cardiovascular events. Composite comprised of myocardial infarction, atrial fibrillation, congestive heart failure, and stroke.

¥ composite comprised of reintubation, non-invasive positive pressure ventilation, and mechanical ventilation.

* adjusted for Elixhauser, age, BMI, ASA class, and procedure.

** adjusted for age, BMI, ASA class

**Table 5.** SSc cohort-specific features of pulmonary hypertension (n=258)

Echocardiographic Evidence of Elevated PA pressure (eRVSP)*	
Elevated Right Ventricular Systolic Pressure (≥40 mmHg), n (%)	31 (12.0%)
Diffuse Cutaneous Variant (N=51)	11 (21.6%)
Limited Cutaneous Variant (N=173)	15 (8.7%)
Overlap Syndrome (N=31)	5 (16.1%)
Sine Variant (N=3)	0 (0%)
Right Ventricular Systolic Pressure, mean (SD)	60.0 (±18.7)
**Diagnosed Pulmonary Hypertension (PH)** **	
Pulmonary Hypertension (PAMP≥25) n (%)**	22 (8.5%)
Diffuse Cutaneous Variant (N=51)	6 (11.8%)†
Limited Cutaneous Variant (N=173)	12 (6.9%)
Overlap Syndrome (N=31)	4 (12.9%)
Sine Variant (N=3)	0 (0%)
Pre-capillary PH (pulmonary wedge pressure <15mmHg)	15 (68.2%)
Diffuse Cutaneous Variant (N=51)	4 (7.8%)†
Limited Cutaneous Variant (N=173)	10 (5.7%)
Overlap Syndrome (N=31)	1 (3.2%)
Sine Variant (N=3)	0 (0%)
Post-capillary PH (pulmonary wedge pressure >15mmHg)†	7 (31.8%)
Diffuse Cutaneous Variant (N=51)†	2 (3.9%)†
Limited Cutaneous Variant (N=173)	2 (1.2%)
Overlap Syndrome (N=31)	3 (9.7%)
Sine Variant (N=3)	0 (0%)
**Detailed Right Heart Catheterization Data mean (SD)**	
Right Atrial Pressure (in mmHg)	8.1 (±3.3)
Right Ventricular Systolic Pressure (in mmHg)	58.9 (±13.1)
Right Ventricular Diastolic Pressure (in mmHg)	6.8 (±5.9)
Pulmonary Artery Systolic Pressure (in mmHg)	61.2 (±14.8)
Pulmonary Artery Diastolic Pressure (in mmHg)	24.6 (±9.1)
Pulmonary Artery Mean Pressure (in mmHg)	39.1 (±9.9)
Pulmonary Artery Wedge Pressure (in mmHg)	12 (±5.4)
Cardiac Index (L/min/m^2^)	5.3 (±1.6)
Pulmonary Vascular Resistance (Wood Units)	5.4 (±4.2)

* by transthoracic echocardiography within 1 year of surgical encounter (% of entire SSc cohort).

** by right heart catheterization within 2 years of surgical encounter (% of entire SSc cohort).

† % describes proportion w/in variant type

## Abbreviations

ASA: American Society of Anesthesiologists
BMI: body mass index
CKD: chronic kidney disease
CHF: congestive heart failure
CI: 95% confidence interval
CREST: calcifications, Raynaud’s phenomenon, esophageal hypomotility, sclerodactyly, and telangiectasias
DVT/PE: deep vein thrombosis/pulmonary embolism
ETT: endotracheal anaesthesia
FOBI: fiberoptic bronchoscopy intubation
ICD-10: International Classification of Disease
ILD: interstitial lung disease
LMA: laryngeal mask airway
NSAID: non-steroidal anti-inflammatory drugs
OR: odds ratio
p: p-value
PAH: pulmonary arterial hypertension
PH: pulmonary hypertension
RSI: rapid sequence intubation
SD: standard deviation
SSc: systemic sclerosis/scleroderma
SLE: systemic lupus erythematosus
