# Supplementary material for: Perioperative Complications in Patients with Systemic Sclerosis: A Comparative Cohort Analysis
Source: Med Res Arch. Author manuscript; Available in PMC 2023 Nov 30. (PMC10688569; doi:10.18103/mra.v11i10.4606)
Supplement: 1 [file NIHMS1944740-supplement-1.pdf]

Supplementary Table 1. Systemic sclerosis-specific cohort characteristics.

Supplementary Table 2. Detailed description of systemic sclerosis surgical site infections.

	**Diffuse Cutaneous** [N=51 (19.8%)]	**Limited Cutaneous** [N=173 (67.1%)]	**Overlap Syndrome** [N=31 (12.0%)]	**Sine** [N=3 (1.2%)]	**Not Recorded** (of total N=258)‡
**Pre-procedural Smoking History n (%)†**					
Active smoker	0 (0%)	9 (5.2%)	1 (3.2%)	1 (33.3%)	
Former smoker	25 (49%)	69 (39.9%)	19 (61.2%)	1 (33.3%)	
Nonsmoker	22 (43.1%)	91 (52.6%)	11 (35.5%)	1 (33.3%)	
Not Recorded	4 (7.8%)	4 (2.31%)	0 (0%)	0 (0%)	
**Select Pre-procedural Medications n (%)†**					
Corticosteroid (any)	13 (25.4%)	147 (85.0%)	23 (74.2%)	1 (33.3%)	
Nonsteroidal Anti-inflammatory	12 (23.5%)	47 (27.2%)	10 (32.3%)	1 (33.3%)	
Immunosuppressant (excl. steroid)	31 (60.7%)	40 (23.1%)	16 (51.6%)	1 (33.3%)	
Anticoagulant	3 (5.8%)	15 (8.7%)	2 (6.5%)	0 (0%)	
Antiplatelet	10 (19.6%)	45 (26.0%)	8 (25.8%)	1 (33.3%)	
Anti-reflux					
None	13 (25.4%)	63 (36.4%)	9 (29.0%)	0 (0%)	
Proton pump inhibitor	35 (68.6%)	93 (53.8%)	20 (64.5%)	3 (100%)	
H2 antagonist	2 (3.9%)	7 (4.0%)	2 (6.5%)	0 (0%)	
Other	1 (2.0%)	10 (5.8%)	0 (0%)	0 (0%)	
**Pre-procedural Antibody**					
Antibody Positivity (any)	32 (59.6%)	124 (71.6%)	25 (80.6%)	2 (66.7%)	57 (22.1%)
Scl-70	16 (31.3%)	11 (6.3%)	2 (6.4%)	2 (66.7%)	46 (17.8%)
Antinuclear Antibodies (ANA)	31 (%)	120 (69.3%)	22 (70.9%)	2 (66.7%)	46 (17.8%)
Anticentromere Antibody (ACA)	3 (5.8%)	45 (26.0%)	1 (3.2%)	0 (0%)	47 (18.2%)
Mitochondrial (M2) Antibody	0 (0%)	2 (1.1%)	3 (9.6%)	0 (0%)	48 (18.6%)
u1rnp Antibody	2 (3.9%)	3 (1.7%)	2 (6.4%)	0 (0%)	52 (20.2%)
**Pre-procedural Associated Autoimmune Disorders n (%)†**					
None	12 (22.0%)	33 (19%)	0 (0%)	0 (0%)	n/a
Rheumatoid Arthritis	6 (11.7%)	9 (5.2%)	11 (35.4%)	0 (0%)	n/a
Systemic Lupus	1 (1.9%)	2 (1.2%)	8 (25.8%)	0 (0%)	n/a
Raynaud's Phenomenon	31 (60.7%)	120 (69.3%)	6 (19.4%)	1 (33.3%)	n/a
Antiphospholipid Syndrome	0 (0%)	1 (0.6%)	0 (0%)	0 (0%)	n/a
Sarcoidosis	0 (0%)	4 (2.3%)	2 (6.4%)	2 (66.7%)	n/a
Sjogren's Syndrome	1 (1.9%)	3 (1.7%)	3 (9.7%)	0 (0%)	n/a
Multiple Sclerosis	0 (0%)	1 (0.6%)	0 (0%)	0 (0%)	n/a
Myositis	0 (0%)	0 (0%)	1 (3.2%)	0 (0%)	n/a
**Pulmonary Function Test n (%) or mean (SD) within one year of procedure†**					
Any PFT	23 (45.1%)	45 (26.0%)	13 (41.9%)	2 (66.7%)	175 (61.4%)
Any PFT	2.9 (±0.8)	3.28 (±1.0)	2.8 (±1.0)	4.5 (±0.3)	
Any PFT	72.4 (±19.3)	90.0 (±22.1)	75.6 (±22.3)	75.5 (±24.7)	
Any PFT	2.3 (±0.7)	2.5 (±0.6)	2.2 (±0.8)	3.6 (±0.1)	
Any PFT	71.8 (±19.1)	84.4 (±21.3)	74.3 (±20.2)	70.5 (±26.2)	
Any PFT	3.5 (±1.0)	4.9 (±1.5)	3.9 (±0.7)	5.2 (±0.1)	
Any PFT	67.6 (±15.3)	87.4 (±18.4)	92.5 (±14.8)	80.1 (±19.3)	
Any PFT	12.7 (±6.5)	17.7 (±6.0)	11.5 (±3.5)	20 (±6.4)	
Any PFT	62.4 (±26.8)	65.1 (±24.8)	46.3 (±28.3)	62.1 (±25.4)	
**Selected Sub-cohort Univariate and Mulivariable**					P-value¥
**Pre-procedural Individual**					
Pre-procedural Interstitial Lung Disease	34 (66.7%)	37 (21.3%)	13 (41.9%)	1 (33.3%)	**<0.001**
Pre-procedural OSA	7 (13.7%)	30 (17.3%)	5 (16.1%)	0 (0%)	0.8
Pre-procedural Home Oxygen Use	11 (21.5%)	6 (3.5%)	3 (9.6%)	0 (%)	**<0.001**
**Airway Characteristics & Management†**					
Microstomia	37 (72.5%)	11 (6.4%)	6 (19.3%)	0 (0%)	**0.02**
Limited Neck Range of Motion	12 (23.5%)	20 (11.6%)	3 (9.7%)	0 (0%)	0.26
Ventilation Grade 3 or 4	0 (0%)	0 (0%)	1 (1.1%)	0 (0%)	0.39
Direct laryngoscopy View 3 or 4	1 (1.1%)	4 (2.3%)	1 (3.2%)	0 (0%)	0.89
Video Laryngoscopy*	5 (5.6%)	20 (22.2%)	2 (2.2%)	0 (0%)	0.77
**Selected Clinical Outcomes**					
**Intraoperative Complications****					
Aspiration	0 (0%)	3 (1.7%)	0 (0%)	0 (0%)	0.68
Induction-related bradycardia	10 (19.6%)	25 (14.5%)	7 (22.5%)	0 (0%)	0.51
Arrhythmia	1 (2%)	5 (2.9%)	0 (0%)	0 (0%)	0.78
New Intensive Care Unit Admission	4 (7.8%)	9 (5.2%)	1 (3.2%)	0 (0%)	0.75
**Selected In-hospital outcomes†**					
Postoperative Day 0-7 Delirium	1 (2.0%)	5 (2.9%)	0 (0%)	0 (0%)	0.8
Postoperative Day Ileus >3 days	4 (7.8%)	8 (4.6%)	0 (0%)	1 (33.3%)	0.06
**30-day Postoperative Complications****					
Major Adverse Cardiovascular Events	4 (7.8%)	11 (1.9%)	0 (0%)	0 (0%)	0.46
Major Infection Composite (total)	4 (7.8%)	20 (11.5%)	5 (16.1%)	0 (0%)	0.63
Endoscopic	2 (3.9%)	3 (1.7%)	0 (0%)	0 (0%)	
Non-endoscopic	2 (3.9%)	17 (9.8%)	5 (16.1%)	0 (0%)	**0.04**
Surgical Site Infection	1 (2%)	5 (2.9%)	3 (9.6%)	0 (0%)	0.25
30-day pneumonia, any type	1 (1.9%)	7 (4.0%)	2 (6.4%)	0 (0%)	0.76

Abbreviations: n/a: not applicable; H2 antagonist: histamine type-2 receptor antagonist; u1rnp antibody: anti-U1 ribonucleoprotein autoantibody; PFT: pulmonary function test; FVC: forced vital capacity; FEV1: forced expiratory volume, 1st second; TLC: total lung capacity; DLCO: diffusing capacity for carbon monoxide; CO: carbon monoxide; OSA: obstructive sleep apnea

‡% refers to total proportion of registered values

* % refers to total general endotracheal anesthesia cases (N=90) in SSc cohort

** % refers to total proportion of registered values

¥ p value describes between group differences
